# UMTS Base Station-like Exposure, Well-Being, and Cognitive Performance

**DOI:** 10.1289/ehp.8934

**Published:** 2006-06-06

**Authors:** Sabine J. Regel, Sonja Negovetic, Martin Röösli, Veronica Berdiñas, Jürgen Schuderer, Anke Huss, Urs Lott, Niels Kuster, Peter Achermann

**Affiliations:** 1 Institute of Pharmacology and Toxicology, University of Zürich, Zürich, Switzerland; 2 Department of Social and Preventive Medicine, University of Bern, Bern, Switzerland; 3 IT’IS Foundation for Research on Information Technologies in Society, Swiss Federal Institute of Technology, Zürich, Switzerland; 4 Center for Integrative Human Physiology, University of Zürich, Zürich, Switzerland

**Keywords:** base station, cognitive function, electromagnetic hypersensitivity, human exposure, mobile phones, RF EMF

## Abstract

**Background:**

Radio-frequency electromagnetic fields (RF EMF) of mobile communication systems are widespread in the living environment, yet their effects on humans are uncertain despite a growing body of literature.

**Objectives:**

We investigated the influence of a Universal Mobile Telecommunications System (UMTS) base station-like signal on well-being and cognitive performance in subjects with and without self-reported sensitivity to RF EMF.

**Methods:**

We performed a controlled exposure experiment (45 min at an electric field strength of 0, 1, or 10 V/m, incident with a polarization of 45° from the left back side of the subject, weekly intervals) in a randomized, double-blind crossover design. A total of 117 healthy subjects (33 self-reported sensitive, 84 nonsensitive subjects) participated in the study. We assessed well-being, perceived field strength, and cognitive performance with questionnaires and cognitive tasks and conducted statistical analyses using linear mixed models. Organ-specific and brain tissue–specific dosimetry including uncertainty and variation analysis was performed.

**Results:**

In both groups, well-being and perceived field strength were not associated with actual exposure levels. We observed no consistent condition-induced changes in cognitive performance except for two marginal effects. At 10 V/m we observed a slight effect on speed in one of six tasks in the sensitive subjects and an effect on accuracy in another task in nonsensitive subjects. Both effects disappeared after multiple end point adjustment.

**Conclusions:**

In contrast to a recent Dutch study, we could not confirm a short-term effect of UMTS base station-like exposure on well-being. The reported effects on brain functioning were marginal and may have occurred by chance. Peak spatial absorption in brain tissue was considerably smaller than during use of a mobile phone. No conclusions can be drawn regarding short-term effects of cell phone exposure or the effects of long-term base station-like exposure on human health.

In 2003 a Dutch study on the effects of controlled exposure to mobile communication system radio-frequency electromagnetic fields (RF EMF) at base station intensities on human well-being and cognitive function was published (Zwamborn et al. 2003), hereafter called TNO study (TNO - Netherlands Organization for Applied Scientific Research, Physics and Electronics Laboratory). Effects of two systems were explored: the second-generation Global System for Mobile Communication (GSM) widely used around the world, and its successor, the Universal Mobile Telecommunications System (UMTS), the third generation of mobile networks. Two groups of subjects were investigated, consisting of individuals with and without self-reported health complaints attributed to daily life exposures to RF EMF. Although exposure to GSM-like EMF had no effect at the time-averaged incident electric field (E-field) strength of 0.7 V/m, UMTS-like exposure at an E-field strength of 1 V/m reduced well-being in both groups. No consistent effects on cognitive performance were found. The 3 dB difference of the averaged incident fields was unlikely to have contributed to the different outcome of GSM and UMTS exposure on well-being. The results were hypothesized to be due to the different modulation schemes.

The TNO study was the first to investigate a base station-like exposure and to indicate a reduction in well-being. Regarding the stronger but much more localized exposure by mobile phone handsets, there is an abundant yet controversial body of research on potential nonthermal effects on humans. Data on well-being are inconclusive [Rubin et al. 2006; for a review, see Seitz et al. (2005)], yet various studies identified subtle effects regarding changes in brain activity or influences on cognitive function such as reaction times, working memory, and attention (e.g., Curcio et al. 2005; Freude et al. 2000; Huber et al. 2002, 2005; Hyland 2000; Koivisto et al. 2000b; Krause et al. 2000a). Some of the reported changes (e.g., acceleration of response times in certain cognitive tasks, altered oscillatory activity in the electroencephalogram as a function of time and task), however, were inconsistent and could not be replicated (Haarala et al. 2003; Krause et al. 2004; Preece et al. 2005).

An ongoing debate in RF EMF research and the general public concerns self-reported electromagnetic hypersensitivity (EHS) relating to persons attributing subjective complaints of impaired well-being (e.g., headache, nausea, sleep disturbances) to EMF exposure comprising radio frequency as well as extremely low-frequency fields of domestic power supplies (e.g., National Institute of Environmental Health Sciences 1998; Röösli et al. 2004). To date, no causal link has been found between exposure to mobile phones and EHS symptoms [for a review, see Rubin et al. (2005)], and objective criteria for EHS specification could not be established.

The persisting uncertainty associated with potential adverse health effects of the new UMTS technology, together with its rapidly ongoing implementation, has led to widespread public concern in many countries. We designed the present experiment as a follow-up study to clarify the reliability of the TNO study that was largely debated in the scientific community. Meanwhile, additional follow-up studies have been initiated in Denmark, the United Kingdom, and Japan (Andersen J, Challis L, Watanabe S, personal communications). We used validated measuring instruments and an improved setup yielding better uniformity of exposure, as well as an additional E-field strength (10 V/m) to establish a dose–response relationship. Based on the results reported by Zwamborn et al. (2003), we hypothesized that exposure to UMTS-like radiation would attenuate subjective well-being in both sensitive and nonsensitive subjects, possibly in a dose-dependent manner, but would not affect cognitive performance.

## Materials and Methods

### Study participants

We investigated the effects of UMTS-like EMF in subjects with self-reported sensitivity to RF EMF (*n* = 37) and a reference group without complaints (*n* = 91). Because of noncompliance of three subjects and eight dropouts, the final study group included *n* = 33 sensitive (14 males, 19 females) and *n* = 84 nonsensitive subjects (41 males, 43 females). Both groups were recruited from the general public by advertisement in a local newspaper, by flyers, and from databases of two previous studies with sensitive participants willing to participate in future research projects. Because of a lack of an operational tool for measuring sensitivity to EMF (World Health Organization 2005), criteria for recruitment were based on self-reported sensitivity to RF EMF, that is, purported sensing of RF EMF or afflictions related to RF EMF as emitted by mobile or cordless phones and antennas.

Subjects were contacted by telephone and preselected by a standardized interview. Exclusion criteria comprised pacemakers, hearing aids, artificial cochleas, regular consumption of narcotics or psychoactive drugs in the previous 6 months, smoking, polymorbidity with respect to chronic diseases, pregnancy, a medical history of head injuries and or neurologic/psychiatric diseases, sleep disturbances, and an average consumption of alcohol > 10 drinks/week or of caffeinated beverages amounting to > 450 mg caffeine/day (e.g., approximately three cups of coffee). We also excluded shift workers and persons undertaking long-haul flights (> 3 hr time zone difference) within the last month before the experiment.

On their first appointment, all subjects filled in a questionnaire to verify the exclusion and matching criteria (age in decades, sex, and residential area). The entire reference group was frequency matched to the sensitive group, and a subgroup was 1:1 matched, also including body mass index (BMI). Subjects were between 20 and 60 years of age (mean ± SD, 37.7 ± 10.9), right-handed (Oldfield 1971), and of normal body weight (BMI 19–30 kg/m^2^). They gave their written informed consent and were reimbursed for participating. The cantonal ethical committee of the Canton Zürich approved of the study protocol.

### Study design

We performed the study at the Institute of Pharmacology and Toxicology, University of Zürich, between 1 February and 20 May 2005. It consisted of three experimental sessions at 1-week intervals (± 1 day) that were preceded by a training session 7 ± 1 days ahead and that were always scheduled at the same time of day (~ ± 2 hr). Subjects were evenly distributed across experimental period, weekdays, and time of day. We asked them to abstain from any medication 24 hr before each session and also requested them not to use a mobile or cordless phone for 12 hr preceding the sessions.

Exposure was computer controlled providing double-blind conditions, which we applied in a randomized crossover design. Before and after exposure, subjects filled in the questionnaires in an office room and were then escorted to the exposure chambers. Exposure took place in two identical and specially adapted but separate rooms with constant temperature and light conditions. We randomly assigned pairs of subjects to one of six possible sequences of the three exposure conditions [0 (sham), 1, 10 V/m] but shifted the subjects in each pair by 20 min to minimize contact between them. Each exposure session lasted 45 min, during which subjects performed two series of cognitive tasks (sessions 1 and 2), starting at the beginning and after 22 min of exposure, respectively. Between sessions, subjects remained in front of the computer and were allowed to read magazines.

### Exposure and dosimetry

**Figure 1:**
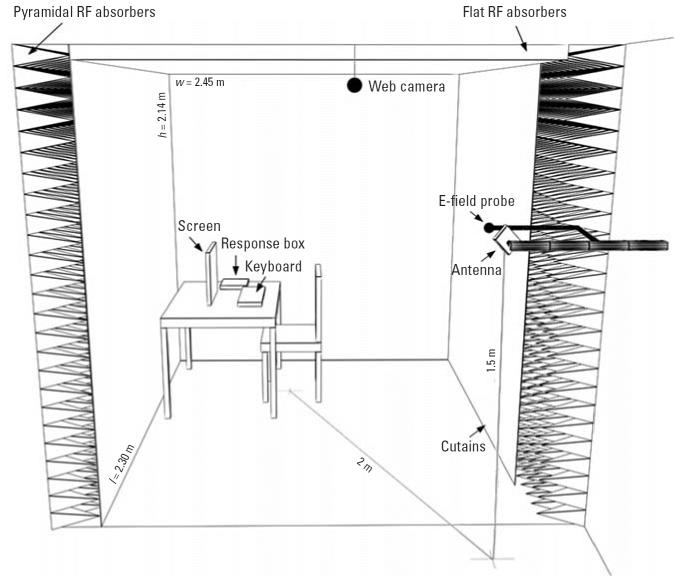
Sketch of the exposure chamber. Walls were covered by pyramidal RF absorbers and nonreflecting curtains. The ceiling was covered by flat absorbers. Antenna, E-field probe, furniture, screen, keyboard, response box, web camera, inner dimensions (*w*, width; *h*, height; *l*, length), and position of the antenna are indicated.

Each experimental room included an exposure area installed as a one-side-open chamber shielded with RF radiation absorbers (Figure 1). We placed the antenna (SPA 2000/80/8/0/V; Huber & Suhner, Herisau, Switzerland) at 1.5 m height and 2 m distance from the subjects, targeting the left side of the body from behind, with a field incidence angle of 25° with respect to the ear-to-ear vertical plane (Figure 1). To produce the same polarization as in the TNO study, we tilted the antenna and thus the E-field 45° from vertical. The antenna possessed a –3-dB beam width of approximately 75° in horizontal and vertical directions, resulting in a uniform E-field distribution in a manner similar to that of the far field of a base station. We verified field uniformity before and after the experimental phase by scanning the exposure area with a field probe. The UMTS signal format was identical to the one used by Zwamborn et al. (2003), consisting of four control and synchronization channels (primary synchronization channel, –8.3 dB below total RF power; secondary synchronization channel, 8.3 dB; primary common control physical channel, –5.3 dB; common pilot channel, –3.3 dB) with a center frequency of 2,140 MHz and chip rate of 3.84 microchips/sec. The signal, generated by a commercial generator (E4433B Options 200, 201, UN8, UN9; Agilent Technologies, Palo Alto, CA, USA), corresponded to a UMTS base station frequency division duplex mode downlink configuration with no active voice calls. Exposure was continuously monitored and regulated (three-axis E-field probe). Each chamber was equipped with a wooden table and chair, a flat-panel monitor with keyboard, a plastic response box for the cognitive tasks, and the UMTS antenna with a field probe (Figure 1). The web camera that recorded the subjects from top left (1 frame/sec) and the computer hardware were outside the exposure chamber. The sum of all magnetic fields (frequency range, 30 Hz–400 kHz) was below 0.2 μT. We measured background RF radiation levels (80 MHz–4 GHz) before and after the experiment, and they remained below 1 mV/m over the whole exposure area.

We conducted numerical dosimetry according to Kuster and Schönborn (2000) using the finite-difference time-domain simulation platform Semcad X (SPEAG, Zurich, Switzerland) and three whole-body anatomical phantoms (two male, one female). We treated reflections from furniture as uncertainty, reducing the computational space to 2.6 × 1 × 1.8 m^3^ (length × width × height). We modeled the floor as concrete (i.e., relative permitivity of 7.5, and conductivity of 0.12 Siemens per meter), whereas the walls and ceiling were modeled as perfectly absorbing boundaries. The numerical discretization of the chamber was 5 × 5 × 5 mm^3^; of the human model, 2 × 2 × 2 mm^3^; and of parts of the antenna, 1 × 0.5 × 1 mm^3^, resulting in approximately 335 million voxels.

The sources contributing to the absolute uncertainty of the average dosimetry were *a*) antenna modeling; 0.1 dB (experimentally verified); *b*) deviation of incident field exposure with respect to the target field including transfer calibration, sensor linearity, feedback control, and reflections from furniture, 0.7 dB; and *c*) average anatomy, dielectric parameters, and discretizations. The variation as a function of weight, sex, and position was assessed separately by scaling the three phantoms in the range of our subjects (47–110 kg; head tissues were based on nonscaled phantoms) and by rotating the phantoms ± 25° around their axis. Because of good uniformity of the field, we could neglect the effect of movement.

### Questionnaires

The short Questionnaire on Current Disposition (QCD) (Müller and Basler 1993) measures subjective well-being within short test–retest intervals using six bipolar items (tense–calm, apprehensive–unperturbed, worried–unconcerned, anxious–relaxed, skeptical–trusting, uneasy–comfortable) and was applied before and after each experimental condition. Outcomes of the QCD comprise the difference between post-and preexperimental scores (QCD_diff_) as well as postexperimental scores (QCD_post_).

We used the modified Quality-of-Life Questionnaire (Zwamborn et al. 2003), henceforth referred to as the TNO-Q, as a reference questionnaire for comparison with the TNO study. The validated, original questionnaire had been developed to estimate “quality of life” during trials of an antihypertensive drug treatment (Bulpitt and Fletcher 1990) and was modified by Zwamborn et al. (2003) by using a selection of 23 items separated in five subscales (anxiety, somatic symptoms, inadequacy, depression, hostility).

We applied a self-designed Questionnaire to include Other Factors (QOF) potentially related to well-being [sleep duration, quality of previous night, suffering from a cold, amount of alcohol and caffeine consumed and medication taken on the day of the experimental session, (pre-)menstrual complaints, and stressful events]. Moreover, subjects had to rate the perceived field strength of the same day’s exposure condition on a visual analogue scale ranging from “not at all” (0) to “very strong” (100 mm). We applied the TNO-Q and the QOF after each experimental condition. Completion of all questionnaires took 5–15 min.

One week before the training and 1 week after the last session, we applied a paper version of the Bern Questionnaire on Well-being (BQW) (Grob 1995). It measures well-being over a few weeks [39 items separated into two main scales (satisfaction, ill health)] and was used to assess whether participation per se had an influence on well-being, regardless of exposure.

### Cognitive tasks

We investigated the effects of UMTS-like radiation on brain functioning with the Simple Reaction Time Task (SRT) and Two-Choice Reaction Time Task (CRT) (Koivisto et al. 2000b; Preece et al. 1998, 1999), the N-back Task (N-back) (Koivisto et al. 2000a), and the Visual Selective Attention Task (VSAT) adapted from Zwamborn et al. (2003) and applied the tasks in fixed order (SRT, CRT, 1-, 2-, 3-back, VSAT). We implemented the tasks using software from e-Prime (Psychology Software Tools Inc., Pittsburgh, PA, USA). We instructed subjects to respond as quickly and accurately as possible by using their right index (targets) and middle (nontargets) finger. Completion of one series took 15–20 min.

In the SRT, a “0” appeared on screen until the subjects pressed the corresponding “0” button on the response box. In the CRT, either “JA” (yes) or “NEIN” (no) was shown, and subjects had to press the “J” (targets) and “N” button (nontargets).

In the N-back, single consonants were randomly presented. Subjects had to compare each current letter with any letter presented one, two, or three trials back (1-, 2-, 3-back, respectively) and press “J” for same letters and “N” for different letters.

In the VSAT, a random combination of four letters and/or crosses in a square was presented. The targets were “U” and “F” appearing on the diagonal from upper left to lower right. Subjects had to press “J” when one or both targets appeared and “N” when no target was presented.

### Statistical analysis

We used linear mixed models for statistical analyses (questionnaires: STATA 9.0, StataCorp, College Station, TX, USA; cognitive tasks: SAS version 8.2; SAS Institute Inc., Cary, NC, USA). With respect to reaction times, we excluded individual outliers over all sessions according to a robust rejection-estimation procedure (4 × median deviation) (Hampel 1985). We transformed reaction times (1/reaction time), which are referred to as speed [1/sec; correct responses only], and checked residuals for normal distribution.

We performed stratified analyses for the sensitive and nonsensitive groups by using a random intercept model presuming an identical intraclass correlation for all subjects. The base model included the factor condition (sham, 1, 10 V/m) and week (1, 2, 3) to account for possible order effects. The model for cognitive data also contained session (session 1, session 2) as a factor and corresponding interaction effects. We modeled condition as a continuous variable to test for a dose–response relationship and assessed differences between groups with an overall model including the factor sensitivity and a sensitivity × condition interaction. We evaluated the robustness of results by adjusting the model for potential confounding factors (Tables 1, 2).

We used the percentage of correct answers in the CRT, 1-, 2-, 3-back and VSAT as a measure of accuracy. Except for the 3-back data, residuals were not normally distributed, and differences were assessed using non-parametric Wilcoxon signed-rank tests. We performed comparisons of 1 V/m versus sham and 10 V/m versus sham for session 1, session 2, and the difference between the two sessions. The resulting *p*-values were adjusted for multiple testing (six tests) according to Bonferroni-Holm (Holm 1979).

To generally control for multiple testing, we performed a multiple end point adjustment for the cognitive outcomes using the method proposed by Tukey et al. (1985).

We analyzed the ability to perceive EMF by calculating Spearman rank correlations between perceived field intensity and true exposure status for each subject. We tested the number of positive and negative correlations using a sign test and used the same procedure to evaluate the association between perceived field intensity and well-being (QCD, TNO-Q).

## Results

### Questionnaires

Well-being as measured by the QCD and the TNO-Q was not affected by exposure (Table 1). With respect to the six items in the QCD and the five subscales of the TNO-Q, we found no significant exposure–response associations in any of the two groups. Regardless of the actual condition, sensitive subjects generally reported more health problems, particularly in the TNO-Q. Neither group showed a relationship between perceived field intensity and true exposure status (Table 1). Sensitive subjects indicated higher field strengths in all conditions (*p* < 0.001), even though score values were not associated with exposure levels. Seventeen of 31 sensitive subjects had a positive correlation between perceived and real field intensity, and 13 had a negative correlation (nonsensitive group, 22 and 27 of 57 subjects, respectively), which can be expected by chance (Table 3). Regardless of exposure condition, perceived field intensity was positively correlated with impaired well-being in 68% of sensitive (QCD_diff_, *p* = 0.043) and 64% of nonsensitive (*p* = 0.001) subjects. Similar results were found with respect to the QCD_post_ and the TNO-Q (data not shown).

In the BQW, comparison of scores 1-week before and after study participation showed no significant changes for satisfaction and ill health in the sensitive group. In the nonsensitive group, the score for ill health was lower after the experiment (*p* = 0.004), but satisfaction remained unchanged.

### Cognitive tasks

In the course of the entire study, subjects got faster in all tasks (*p* < 0.02) except the SRT. In both groups and irrespective of condition, speed decreased significantly from session 1 to session 2 in both the SRT and CRT but increased in the 1-, 2-, 3-back and VSAT (*p* < 0.0001). In the following, only effects including condition or a condition × session interaction are described.

In both groups, we observed no condition-induced effects on speed in the SRT, 1-, 2-, 3-back and VSAT. In the CRT, speed decreased in the sensitive group from session 1 to session 2 in the sham and 1 V/m condition (~ 20 msec) but not in the 10 V/m condition (condition × session, *p* = 0.007; Table 2). In contrast, we observed a decrease in speed between sessions irrespective of exposure condition in the nonsensitive group (*p* = 0.254; Table 2). A mixed-model analysis of variance including the factor sensitivity (sensitive, non-sensitive) corroborated the observed differences between groups with respect to exposure (condition × sensitivity, *p* = 0.005).

Accuracy was not affected by exposure in a dose–response manner in any of the cognitive tasks except the 1-back task in the nonsensitive group, where it decreased from 98.2% (sham) to 97.3% (10 V/m; *p* = 0.046) in session 1.

Adjusting the models for potential confounding factors (Tables 1, 2) or performing the analyses with only the 1:1 matched subjects did not alter the results. After multiple end point adjustment ( α = 0.05; number of tests = 44; overall correlation among cognitive outcomes = 0.39), however, all reported *p*-values exceeded the significance level of *p* = 0.0051 (Tukey et al. 1985).

### Dosimetry

**Figure 2:**
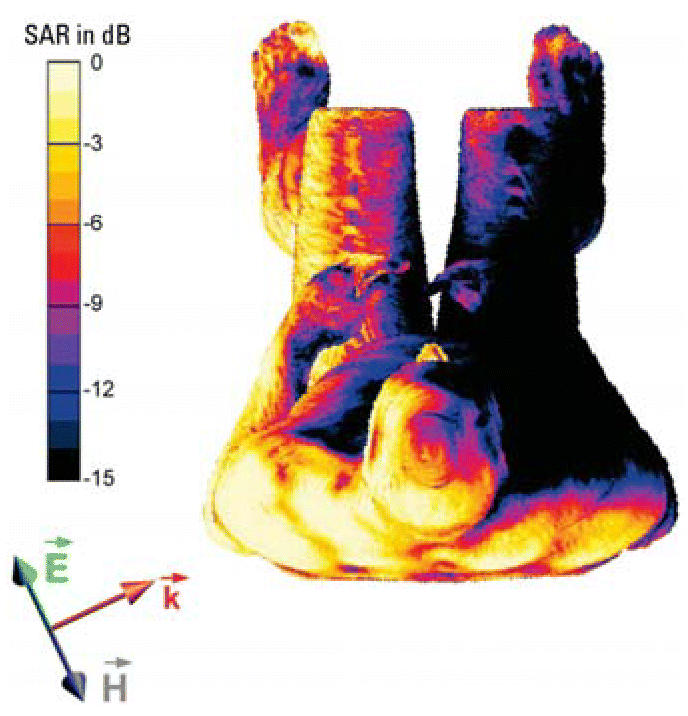
SAR distribution on the surface of a male (80 kg) in a sitting position (top view): 0 dB corresponds to 0.05 W/kg for an E-field strength of 1 V/m. The orientation of the E-field (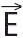), the magnetic field (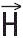), and the propagation direction (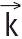) of the EMF are indicated.

Penetration depth was low, and highest specific absorption rate (SAR) values occurred predominantly at the illuminated side close to the skin (Table 4, Figure 2). Whole-body average absorption was 6.2 ± 1.8 and 620 ± 180 μW/kg for 1 V/m and 10 V/m, respectively, with an absolute uncertainty of 41% (Table 4). Peak spatial SAR (averaged over 10 g) was 45 ± 13 and 4,500 ± 1,300 μW/kg, respectively, for brain tissue. At 10 V/m, all values were at least 100 times below recommended safety limits (International Commission on Non-Ionizing Radiation Protection 1998). Compared with use of a mobile phone at the ear or exposure levels used in other studies, the peak spatial SAR of the brain was > 100 times lower at 10 V/m in our study. SAR values for head tissues and left–right differences are shown in Table 5.

The SAR values are strongly dependent on the incidence angle and the polarization of the field that were fixed in our study. Variation of incidence angle and polarization at the same field strength will lead to considerable changes of the SAR values in different parts of the body.

## Discussion

In contrast to our hypothesis, well-being as assessed by the QCD and TNO-Q questionnaires was not affected by UMTS radiation, either in the 1 V/m or in the 10 V/m condition. Even though sensitive subjects generally reported more health problems, we found no difference overall between the two groups with respect to the applied field conditions. Similarly, cognitive performance was not affected except for two separate and marginal effects in the 10 V/m condition. In the CRT we could not observe a slight decrease in speed across sessions in sensitive subjects as observed in the 0 V/m and the 1 V/m condition, and in the 1-back task accuracy was reduced in nonsensitive subjects compared to the sham condition.

Cognitive tasks with moderate to high workload frequently have been used as a tool to assess RF EMF effects on brain physiology by measuring simple motor responses requiring selective attention and higher cognitive functions such as working memory (e.g., Krause et al. 2000b). Except for the VSAT, which was taken from the TNO battery of cognitive tasks for follow-up reasons, we chose the SRT, CRT, and N-back on the basis of recently published work attempting to assess EMF-induced changes with respect to brain physiology (Koivisto et al. 2000a, 2000b; Preece et al. 1999). However, the described effects showed no consistent picture and could not be replicated (Haarala et al. 2003; Preece et al. 2005).

In general, exposure in these studies was poorly defined, and the inconsistencies in cognitive outcome may be due to differences in the design, blinding, study population, and sample size, thus preventing a comparison of the results. Alternatively, cognitive tasks used so far may not be sensitive enough to reliably measure potential RF EMF effects on brain functioning, leading to a masking of existing effects or resulting in significant effects of tests that stochastically respond to RF EMF. Moreover, statistical analysis of several tests increases the risk of false-positive findings.

In the present study, speed was affected in the sensitive group in one of six cognitive tasks and accuracy in the nonsensitive group in one of five tasks. Although we cannot exclude an actual condition × session interaction in the CRT in sensitive subjects and, similarly, a condition effect in the 1-back task in nonsensitive subjects, the findings seem to be coincidental because they did not reach significance after multiple end point adjustment.

Both the sensitive and the nonsensitive groups were unable to identify the applied fields better than expected by chance. Because we investigated only three conditions per subject, the likelihood of correct field rating by chance was relatively high. The observed distribution of 39 individuals with a positive correlation between the applied and estimated exposure conditions and 40 individuals with a negative correlation was likely to be expected by chance. Nevertheless, we cannot exclude that among these subjects a minority was actually able to perceive the applied exposure. The identification of such individuals has failed in several provocation studies so far (reviewed by Rubin et al. 2005) and would require a multiple testing approach to reduce the likelihood of a correct rating by chance. Perceived field strength correlated with an impairment of current well-being in both groups irrespective of exposure condition. Also, sensitive subjects rated perceived field strengths higher than did nonsensitive subjects, yet ratings in both groups were not better than expected by chance and not associated with exposure levels. This indicates that sensitive subjects overestimate their ability to better perceive RF EMF than does the general public (Leitgeb and Schröttner 2003).

Our results differ with respect to both well-being and cognitive performance from the results reported by Zwamborn et al. (2003). The TNO-Q is an adapted and not validated version of the original questionnaire (Bulpitt and Fletcher 1990) and was not designed for short retest intervals. Our findings were corroborated by the results of the QCD, a standardized questionnaire that more reliably measures changes in well-being over short test–retest intervals (Müller and Basler 1993). Contrary to the TNO study, we found no significant effect on speed in the VSAT. It was, however, the only task applied in both studies; all other cognitive tasks were distinct. Zwamborn et al. (2003) found other effects with respect to cognitive tasks and exposure conditions (GSM and UMTS), and we also report an effect on speed in one of six tasks and an effect on accuracy in one of five tasks used. No clear picture, therefore, emerges across the two studies showing reproducible effects of exposure condition or cognitive task.

A number of other factors may contribute more generally to the discrepancies between the TNO study and our study. Sample sizes differ substantially (sensitive subjects, 24 vs. 33; nonsensitive subjects, 24 vs. 84). Our reference group was frequency matched to the sensitive group, and a subgroup was 1:1 matched with respect to sex, age, residential area, and BMI. In the TNO study, all conditions in a particular subject were carried out on a single day, whereas we investigated the subjects at the same time of day in weekly intervals to rule out possible circadian and carryover effects. We further controlled circadian influences by a uniform distribution of experimental sessions across the time of day. Carryover effects may lead to an accumulation of RF EMF radiation over time, thus falsifying potential effects of discrete conditions. Furthermore, inclusion of an additional E-field strength of 10 V/m is likely to have contributed to a more reliable assessment of RF EMF effects.

Technical improvements necessitated the modification of the exposure setup used in the TNO study to achieve a more uniform and reproducible base station-like exposure. Although the signal (carrier frequency and modulation) and the angle of incidence were identical, the spatial incident field distribution was less uniform in the TNO study, where a narrow exposure beam of only 5° width was used, resulting in a larger variation because of differences in height and position of the subjects. In addition, the whole-body exposure conditions applied in this study correspond better to a base-station exposure scenario. However, exposure of head tissues was equivalent in both studies, even though we had a smaller intersubject variability. Further insights regarding the discrepancies between the present and the Dutch study might be gained from other follow-up studies under way in Denmark, the United Kingdom, and Japan, which are also investigating the effect of UMTS base station-like radiation on well-being and cognitive function (Andersen J, Challis L, Watanabe S, personal communications).

In summary, we found no causal relationship between RF EMF and a decrease in well-being or adverse health effects under the given exposure conditions but cannot exclude an effect of UMTS-like EMF on brain functioning. The described effects were weak and not consistent in the two groups of sensitive and nonsensitive subjects. Regarding the implications for public health because of widespread exposure in the living environment, no conclusions about long-term effects of UMTS base station-like EMF can be drawn from the present study, since only a short-term exposure was applied.

## Figures and Tables

**Table 1:** Results of applied questionnaires (mean scores ± SD; *n* = 33 sensitive and *n* = 84 nonsensitive subjects).

Outcome	Group	Sham	1 V/m	10 V/m	Cond.^a^*p*-Value	Cond.^b^*p*-Value
QCD_diff_	Sensitive	0.30 ± 0.83	0.24 ± 0.99	0.24 ± 0.95	0.88	0.95
	Nonsensitive	0.05 ± 0.73	–0.04 ± 0.59	0.02 ± 0.55	0.93	0.95
QCD_post_	Sensitive	2.57 ± 1.06	2.65 ± 1.22	2.61 ± 0.97	0.97	0.96
	Nonsensitive	2.19 ± 0.76	2.05 ± 0.80	2.13 ± 0.78	0.97	0.89
TNO-Q	Sensitive	10.53 ± 9.51	9.61 ± 8.96	9.79 ± 8.38	0.84	0.65
	Nonsensitive	5.23 ± 5.09	4.45 ± 4.92	4.96 ± 5.08	0.78	0.92
Field perception	Sensitive	26.0 ± 31.9	31.2 ± 33.7	29.4 ± 29.7	0.89	0.67
	Nonsensitive	12.9 ± 22.8	5.7 ± 13.1	12.2 ± 23.2	0.24	0.33

A difference score > 0 in the QCD_diff_ corresponds to a degradation in current well-being during the experiment. In the QCD_post_ and the TNO-Q, higher scores refer to a lower well-being. We report only *p*-values of condition (Cond.) (for details, see “Materials and Methods”).

a Adjusted for order.

b Adjusted for order, age, sex, BMI, caffeine intake, medication, (pre-)menstrual complaints, sleep quality, and suffering from a cold.

**Table 2:** Results of cognitive performance (mean speed ± SD).

Outcome	Group	Session	Sham	1 V/m	10 V/m	Cond.^a^,^b^*p*-value	Cond. × session^a^,^b^*p*-value	Cond.^b^,^c^*p*-value	Cond. × session^b^,^c^*p*-value
SRT	Sensitives	1	3.86 ± 0.52	3.78 ± 0.44	3.84 ± 0.48	0.09	0.27	0.07	0.27
		2	3.73 ± 0.56	3.65 ± 0.43	3.78 ± 0.47
	Nonsensitives	1	3.85 ± 0.37	3.85 ± 0.38	3.84 ± 0.43	0.59	0.51	0.37	0.50
		2	3.70 ± 0.44	3.70 ± 0.49	3.68 ± 0.41
CRT	Sensitives	1	2.37 ± 0.28	2.33 ± 0.25	2.33 ± 0.28	0.03	0.01	0.02	0.01
		2	2.25 ± 0.30	2.20 ± 0.27	2.31 ± 0.22
	Nonsensitives	1	2.27 ± 0.26	2.27 ± 0.27	2.24 ± 0.25	0.13	0.25	0.08	0.24
		2	2.22 ± 0.27	2.21 ± 0.27	2.21 ± 0.25
N-back									
1-Back	Sensitives	1	2.15 ± 0.56	2.12 ± 0.55	2.13 ± 0.55	0.90	0.67	0.93	0.67
		2	2.27 ± 0.57	2.29 ± 0.54	2.29 ± 0.49
	Nonsensitives	1	2.12 ± 0.44	2.12 ± 0.48	2.10 ± 0.42	0.57	0.97	0.46	0.98
		2	2.26 ± 0.44	2.28 ± 0.48	2.24 ± 0.43
2-Back	Sensitives	1	1.59 ± 0.46	1.53 ± 0.44	1.53 ± 0.35	0.61	0.44	0.50	0.43
		2	1.70 ± 0.49	1.71 ± 0.53	1.71 ± 0.47
	Nonsensitives	1	1.63 ± 0.39	1.58 ± 0.39	1.60 ± 0.38	0.44	0.52	0.37	0.52
		2	1.74 ± 0.42	1.74 ± 0.43	1.72 ± 0.39
3-Back	Sensitives	1	1.48 ± 0.40	1.48 ± 0.46	1.48 ± 0.39	0.57	0.52	0.39	0.51
		2	1.56 ± 0.42	1.60 ± 0.51	1.54 ± 0.37
	Nonsensitives	1	1.56 ± 0.44	1.57 ± 0.51	1.51 ± 0.36	0.59	0.11	0.64	0.11
		2	1.70 ± 0.55	1.64 ± 0.50	1.70 ± 0.49
VSAT	Sensitives	1	1.74 ± 0.33	1.72 ± 0.31	1.75 ± 0.31	0.28	0.94	0.22	0.94
		2	1.85 ± 0.29	1.85 ± 0.31	1.87 ± 0.28
	Nonsensitives	1	1.69 ± 0.34	1.69 ± 0.33	1.68 ± 0.29	0.64	0.70	0.50	0.71
		2	1.79 ± 0.32	1.83 ± 0.36	1.79 ± 0.31

Mean speed ± SD [1/reaction time (1/sec); *n* = 33 sensitive and *n* = 84 nonsensitive subjects] in the two sessions (first and second half of exposure) in the SRT CRT, N-back, and VSAT. We report only *p*-values of condition (Cond.) and of the interaction condition × session (for details, see “Materials and Methods”). Statistical analysis is based on data of all subjects. Because of a missing session in some subjects, mean values are based on subjects who completed both sessions in each condition (*n* = at least 32 sensitive and *n* = at least 77 nonsensitive subjects).

a Adjusted for order.

b *p*-Values not adjusted for testing multiple end points.

c Adjusted for order, age, sex, BMI, caffeine intake, medication, (pre-)menstrual complaints, sleep quality, and suffering from a cold.

**Table 3:** Correlations between perceived E-field strength and real exposure condition (sham, 1 V/m, 10 V/m).

		Correlation between perceived and real field			
	*n*	Positive	Negative	Zero	*p*-Value^a^
All	88	39	40	9	1
Sensitive	31	17	13	1	0.58
Nonsensitive	57	22	27	8	0.56

Two sensitive and 27 nonsensitive subjects perceived no field in all three conditions and were omitted from the analysis.

a Sign test.

**Table 4:** Averaged SAR for whole body and brain and peak spatial averaged SAR for whole body, brain, skin, and muscle for an E-field strength of 1 V/m.

Tissue	SAR [average ± SD (μW/kg)]	Uncertainty [95% CI (%)]
Whole body	6.2 ± 1.8	41
10 g (peak spatial)	150 ± 49	39
1 g (peak spatial)	320 ± 130	41
Brain	11 ± 2.4	48
10 g (peak spatial)	45 ± 13	45
1 g (peak spatial)	73 ± 16	44
Skin
10 g (peak spatial)	230 ± 48	50
1 g (peak spatial)	380 ± 76	39
Muscle
10 g (peak spatial)	120 ± 31	48
√1 g (peak spatial)	190 ± 62	39

Data are, for an E-field strength of 1 V/m, averaged SAR values ± SD of variations and the absolute uncertainty [95% confidence interval (CI)] for whole body and brain, and peak spatial averaged SAR for whole body, brain, skin, and muscle (1 and 10 g) of an average male (80 kg). To obtain SAR values at a field strength of 10 V/m, SAR values in the table have to be multiplied by 100.

**Table 5:** Ratios of averaged SAR values between various organs or tissue and whole body and between left and right sides.

Organ/tissue	Ratio organ or tissue:whole body	Ratio left:right
Gray matter (left hemisphere)	3.5	2.9
White matter (left hemisphere)	2.0	2.6
Cerebellum	0.52	—
Hippocampus (left hemisphere)	0.84	1.6
Hypothalamus (left hemisphere)	0.52	1.9
Thalamus (left hemisphere)	0.64	0.81
Parotid gland	4.6	—
Ear pinna (left)	17	18
Eyeball (left)	5.6	8.8

Data are ratios, for an average male (80 kg), between organ or tissue averaged SAR values and the whole-body averaged SAR value (6.2 μW/kg at 1 V/m) for regions of the brain, ear, eye and throat and the ratio between the averaged SAR values of the left and right sides. The parotid gland is the largest of the salivary glands and was looked at specifically.
